# Perceptions of Risk and Responses to Tanning Bed Warning Labels: A Pilot Study

**DOI:** 10.1155/2022/1090619

**Published:** 2022-07-21

**Authors:** John M. McGrath, Harry Wallace

**Affiliations:** ^1^Department of Human Communication, Trinity University, San Antonio 78212, TX, USA; ^2^Department of Psychology, Trinity University, San Antonio 78212, TX, USA

## Abstract

Tanning bed use has been linked to increases in skin cancer among young women. Although a causal relationship between ultraviolet radiation emitted by tanning beds and melanoma is well established, it is unclear if tanning bed users are aware of the risk and how they would respond to a warning message. Two hundred and ten women aged 16–29 who had used a tanning bed at least once in the last year were asked about their perceptions of risk and their responses to a warning label. Participants were already aware that tanning beds could cause cancer, but after viewing a warning label, most people said they would stop or reduce their tanning bed use. Reactions to a prototype warning label were encouraging and future research should pursue the possibility that current guidelines for tanning bed warning labels may need to be revised.

## 1. Introduction

Skin cancer is the most prevalent cancer in the world and its incidence has been rapidly increasing in the United States [1]. Diagnoses of new cases of melanoma, the deadliest form, are increasing disproportionally among young women because of their use of indoor tanning beds. One study observing 63 women diagnosed with melanoma before age 30 found that 61 of them (97%) had used tanning beds. [2] Although the link between tanning bed use and skin cancer including melanoma has been well-established [3–5], it is unclear whether tanning bed users are aware of the risk and how they would respond to skin cancer warnings. For example, some studies have shown that tanning bed users are unaware of the risks or have misconceptions, such as believing that indoor tanning bed use is safer than outdoor tanning [6, 7]. Other research found that tanning beds were more likely to be used by young women, the majority of whom were aware of the associate risks [8–10]. Given the increases of melanoma cases among the most frequent users of tanning beds, it is important to find out more about their perceptions of risks and to identify possible messaging strategies that may influence them to reduce or stop their use of tanning beds. In this pilot study, we asked tanning bed users about their perceptions of risk and tested their responses to an explicit tanning bed warning label.

## 2. Method

We used an online software platform and crowdsourced data base, Qualtrics Panels, to screen for women aged 16–29 who had used a tanning bed at least once in the last year in the United States. Crowdsourcing involves soliciting online participation from large and diverse groups of people, who register on a website to complete surveys for which they are paid a small fee. To screen participants, they were asked to give their opinions regarding a variety of consumer products, including exercise equipment, computers, and tanning beds, among other items. If they responded “yes” to having used a tanning bed within the last 12 months, they were prompted to respond to a question about cancer risk and then on the following screen to a depiction of a tanning bed warning label. The rationale for this screening approach was to mitigate potential selection and response bias in crowdsourced surveys that can be related to participants knowing the characteristic of interest in the survey or the purpose of the study [11–13]. Recent studies have demonstrated the validity of crowdsourcing and online panel data collection platforms, including Qualtrics [14, 15]. A total of 210 participants, who met the age and tanning bed use criteria, completed the survey.

One goal here was to pilot test a prototype warning label. This warning label was designed according to the guidelines of the American National Standard Institute's (ANSI's) warning label standard, ANSI Z535.4. ANSI-styled warnings are based on the latest peer-reviewed research on warning labels and have shown often to be more effective than other formats or labels that do not follow ANSI guidelines [16]. Conversely, according to previous research, the content and design of current tanning bed warning labels have not been informed by health communication best practices, are not based on research [17], and may be hard to see or missing [18].

**Figure 1:**
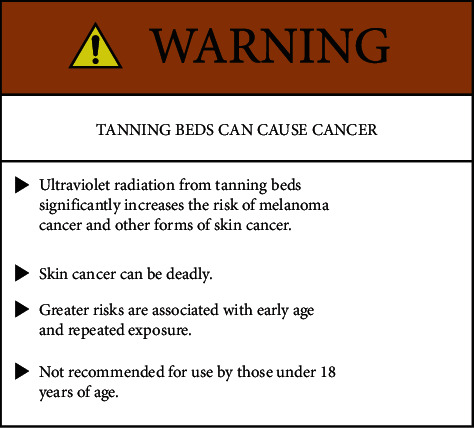
Warning label shown to tanning bed users.

ANSI guidelines recommend utilizing a signal word (i.e., danger, warning, caution) coupled with a safety alert symbol (exclamation mark within a triangle), a specific identification of the risk, how to avoid the risk, and the consequence of not avoiding the risk. The guidelines also recommend a tiered, outlined format, the use of clear, explicit language, and the use of bold and contrasting colors [19].  Figure 1 is the ANSI-styled tanning bed warning label used in this study.

Data were also gathered regarding reactions to specific parts of the warning label, wherein participants were asked to click once on the area of the warning label that caught their attention the most. This study was approved through a university human subjects committee.

## 3. Results

**Figure 2:**
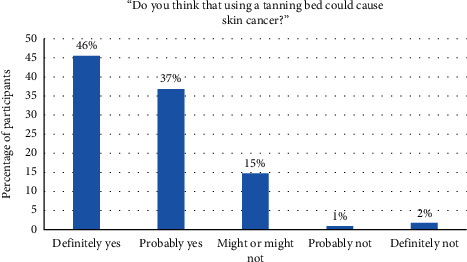
Tanning bed users' perspectives on skin cancer.

**Figure 3:**
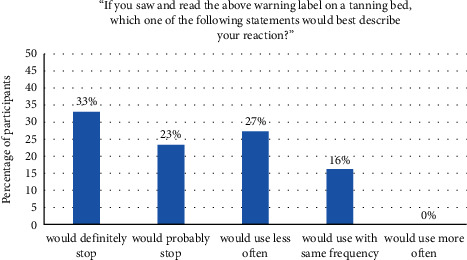
Tanning bed users' reaction to warning label.

Results showed that most tanning bed users were aware that tanning beds could cause cancer (see Figure 2). In fact, 83% of the respondents thought that tanning beds could either “definitely” or “probably” cause skin cancer. However, when shown an explicit warning label, the vast majority of participants said they would “definitely stop,” “probably stop,” or would use tanning beds “less often” (see Figure 3). Participants also indicated which part of the label captured their attention the most. The most frequent response was to the statement, “TANNING BEDS CAN CAUSE CANCER,” which was given by 35% of participants, followed by the statement, “Ultraviolet radiation from tanning beds significantly increases the risk of melanoma cancer and other forms of skin cancer” (20% of participants), and then to the signal word, “WARNING” (17% of participants).

## 4. Conclusion

Indoor tanning beds have been linked to an increased risk of melanoma among young women, and have been classified by the World Health Organization as carcinogenic to humans [20]. In the present study, most tanning bed users were aware that tanning beds could cause cancer, but after viewing an explicit warning label they indicated they would stop or cut down on their tanning bed use. Although intentions do not always align perfectly with future behavior, results suggest that the prototype label at least has the potential to influence users in a way that would reduce their risk of skin cancer. Moreover, it may be possible to improve current tanning bed warning labels that have been described in the literature as being poorly designed and not properly displayed [17, 18]. Tentative conclusions are warranted because we did not compare different labels in this study but several design characteristics can be noted.

Signal words and bold, and contrasting colors serve as an alerting mechanism that calls attention to a label and the seriousness of the message [21, 22]. Explicit language influences peoples' perception of risk [23], and it could be that a specific reference to melanoma had such an effect. Similarly, the inclusion of so called “consequence statements” or outcomes of the hazard tend to capture attention to improve the effectiveness of a warning [24, 25] and in this case, the prominently placed consequence statement, “TANNING BEDS CAN CAUSE CANCER,” was the part of the label that captured the participants' attention the most. Moreover, stronger negative emotions associated with explicit warning labels are more likely to stimulate behavior change [26].

The tanning bed industry has a history of providing false and misleading information to downplay the risk of cancer. In one study, investigators representing themselves as fair-skinned teenage girls contacted 300 tanning salons nationwide in the U.S., including at least 3 in each state. Nearly, all salons denied the known risks of indoor tanning, saying, for example, that the link between indoor tanning and skin cancer is a “big myth.” Four out of five salons falsely claimed that indoor tanning is beneficial to a young person's health [27]. Perhaps consequently, tanning beds remain popular and socially acceptable. For example, nearly half of the gyms in the United States offer indoor tanning [28], and of the top 125 US colleges and universities, 48% had indoor tanning facilities either on campus or in off campus housing [29]. The results of this study suggest that further research is warranted and that if a public health goal is to reduce the risk of cancer through labeling, it may be time to at least consider using more explicit tanning bed warnings that are based on best practices and communication science.

## Data Availability

The data can be available upon request. The requesting party can contact the corresponding author.
